# Biomedical Applications of Humic Substances: From Natural Biopolymers to Therapeutic Agents

**DOI:** 10.3390/antiox14091139

**Published:** 2025-09-21

**Authors:** Yana Gvozdeva, Petya Peneva, Plamen Katsarov

**Affiliations:** 1 Department of Pharmaceutical Technology and Biopharmacy, Faculty of Pharmacy, Medical University of Plovdiv, 4002 Plovdiv, Bulgaria; yana.gvozdeva@mu-plovdiv.bg (Y.G.); petya.peneva@mu-plovdiv.bg (P.P.); 2 Research Institute at Medical University of Plovdiv (RIMU), 4002 Plovdiv, Bulgaria

**Keywords:** humic substances, fulvic acid, humic acid, antioxidant potential, biomedical application

## Abstract

Humic substances, which include humic acid and fulvic acid, are natural biopolymers formed from the decomposition of organic matter. There is growing interest in them because of their diverse potential in the biomedical field. Their complex structures, rich in various functional groups, provide antioxidant, anti-inflammatory, antimicrobial, antiviral, and immunomodulatory properties. Recent studies demonstrate that humic substances can scavenge reactive oxygen species, modulate cytokine production, inhibit viral fusion, promote wound healing, and enhance gut microbiota balance. Humic acid and fulvic acid also exhibit anticancer activity by inducing apoptosis in tumor cells, while protecting healthy tissues from oxidative stress. Furthermore, their chelating capacity underlies detoxifying activity and heavy metal binding effects. Despite promising research, variability in composition and potential cytotoxicity under certain conditions emphasize the need for standardized extraction methods and rigorous preclinical evaluation. This review offers a comprehensive overview of the biological effects of humic substances, exploring the mechanisms behind their actions and their potential biomedical applications. It highlights both the benefits and the limitations associated with their use in drug delivery systems.

## 1. Introduction

In light of the modern trend for a healthy life and increasing its duration, scientists are returning to well-known materials of natural origin in search of specific new properties. Such a material of organic origin, known since the time of the ancient Romans, is humus. The main source of humus formation in the soil is the organic residues of dead plants, animals and microorganisms. The formation of humus is a very complex biochemical process that occurs under the influence of microorganisms. It goes through separate stages: accumulation of organic matter, mineralization and humification. The accumulation stage involves the introduction of plant residues and other organic materials into the soil, during which the organic residues decompose under the action of enzymes secreted by microorganisms. When they come across organic residues, the enzymes first destroy their anatomical structure, and later break down the complex organic substances of which they are made (proteins, sugars, cellulose, lignin, etc.). During mineralization, soil microorganisms break down these materials into simpler compounds, such as carbon dioxide, water, and mineral nutrients. In the final humification, the remaining organic matter is transformed into stable humic components, including fulvic acid (FA), humic acid (HA), and humin (HM) [1,2].

Humic substances (HS) are large, heterogeneous organic molecules that occur naturally in soil, peat, lignite (brown coal), and natural waters. They are formed through the microbial decomposition and chemical transformation of organic matter, including the remains of plants and animals [3]. HS are formed through abiotic combinatorial processes, resulting in supramolecular structures composed of molecules containing varying proportions of carboxylic and phenolic groups [4]. The chemical structure of HS is rich in functional groups—particularly phenolic and carboxyl groups and their derivatives—which influence key properties like water solubility, cation exchange capacity, and mycotoxin binding. Their differing acid–base (protolytic) characteristics enable their separation into distinct fractions. The most acidic fraction, rich in carboxylic groups and soluble in acidic conditions, is known as FA [5]. In contrast, the less acidic fraction, which is richer in phenolic groups and insoluble at low pH, is referred to as HA. It can be further divided into more polar components, such as hymatomelanic acids, which are soluble in ethanol [6]. The diverse and intricate nature of HS also contributes to their polyelectrolyte behavior and chelating capabilities. Over the years, HS have been extensively studied for their detoxifying effects and their potential anti-inflammatory, pro-inflammatory, anticancer, and antiviral activities [3].

HA and FA are the main components of HS with very diverse biomedical applications. The aim of the present work is to analyze and summarize the current achievements in the research of both groups of acids and their biomedical applications.

## 2. Methods

This review is based on articles from PubMed, Web of Science, ScienceDirect, and Google Scholar. The literature search covers a long period from the first articles reporting therapeutic effects for humic substances to 2025, with 111 references selected based on their relevance to the research topic. Keywords such as “humic acid”, “fulvic acid”, “humic substances”, “mechanism of action”, “physicochemical properties”, “biomedical application”, and “drug delivery systems” were used. We conducted a Google search to identify commercial products containing HA and FA. Using the keywords “humic acid”, “fulvic acid”, and “dosage forms”, we found a total of 15 products and their variants, which were summarized.

## 3. Chemical Structure and Physicochemical Properties of HA and FA

**Figure 1:**
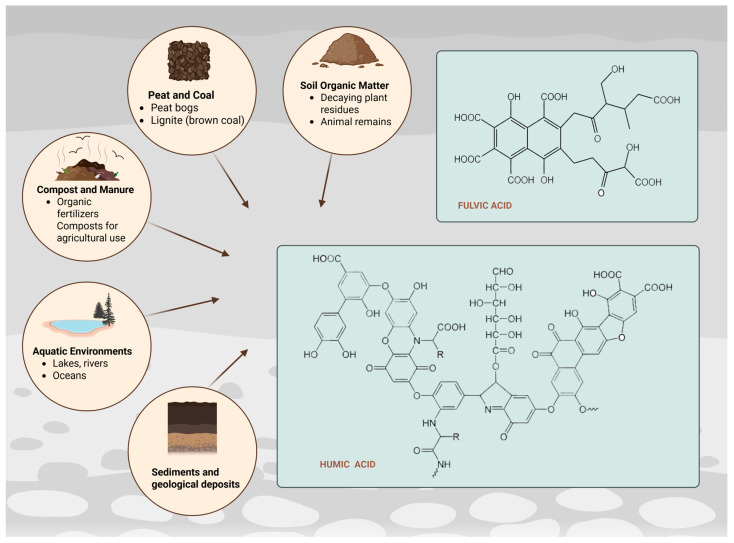
Chemical structure and main sources of humic and fulvic acid (Created in BioRender. https://BioRender.com/26bxqzt, accessed on 23 August 2025).

HA and FA are obtained by a common method of alkaline extraction from soils and sediments (Figure 1). Alkaline extraction could be conducted with sodium hydroxide (NaOH), potassium hydroxide (KOH) and sodium pyrophosphate (Na_4_P_2_O_7_) as alternative [7]. Obtaining HA involves removing insoluble humin microparticles from the products of an alkaline extraction. This is followed by an acidic extraction, during which soluble humates precipitate. The resulting sediments are further purified to yield HA, while the soluble fraction that remains in the acidic solution is referred to as FA [8]. Practically the final highly alkaline extract is acidified, in which the HAs precipitate, while the FAs remain dissolved. FA dissolves at all pH conditions, which is due to their hydrophilic nature. HA dissolves in alkaline media, precipitating at pH < 2. The third fraction of HS–HM is insoluble at all pH conditions [9]. Table 1 summarizes data on the alkaline extraction process from various sources. The conditions of alkaline extraction vary according to the used material and the choice of alkalizing agent.

HA and FA can be described as high-molecular-weight acidic and hydrophilic biopolymers containing carboxyl and phenolic-hydroxyl groups [8]. According to literature biopolymers are polymers obtained from natural sources and often possess biodegradability [11]. HA contain more hydrogen, carbon, nitrogen and sulfur and less oxygen than FA. The increase in molecular weight and carbon content and decrease in oxygen content of HA compared to FA is associated with a change in color from yellow to yellow-brown for FA to dark brown-black for HA [12]. Since humic compounds are usually heterogeneous mixtures of substances, the exact determination of their structure is very controversial. In general, FA have a lower molecular weight than HA [13] (Table 2).

A study by Ukalska-Jaruga et al. (2021) [14] investigated the molecular characteristics of FA and HA obtained from soils with different physicochemical properties. They identified acidic, amino and phenolic functional groups within the aromatic rings and aliphatic chains of humic acids (HAs), whereas fulvic acids (FAs) predominantly exhibited methyl, ethenyl, methylene, and carboxyl groups as their main reactive groups. FAs are characterized by an elliptical shape associated with long aliphatic chains, while HA shows a smaller particle diameter and a more spherical shape as a result of higher intermolecular interactions between particles [14].

The structure of HA is dominated by phenolic and carboxyl groups, which determines the weakly acidic properties of HA. The total acidity of compounds of phenolic and carboxyl groups has been determined to be about 6 meq g^−1^ [15]. On the other hand, hydrophobic moieties represented by aliphatic chains and aromatic rings are also observed in the structure of HA. Briefly, this complex structure possesses amphiphilic character and forms micelle-like structures called pseudo-micelles in neutral to acidic conditions [16,17,18]. Moreover, this structure of HA determines its strong surfactant properties [19,20,21]. These properties can be used as a potential new technology in the study of drug carriers and drug delivery, as well as the development of cosmetic products with various applications.

FAs are more hydrophilic and less polydisperse than HAs. These properties, taken together with their solubility in water at any pH and the polyelectrolyte nature of aqueous solutions, determine the higher chemical and physicochemical activity of FAs [13].

## 4. Relationship Between Structure, Mechanism of Action and Application

The main properties and biomedical applications of HS are related to the structural and functional groups that are found in their composition. As explained in the previous paragraph, HAs contain phenolic and carboxylic functional groups that allow the deprotonation of OH/OOH. This process is associated with the ability of HS to form complexes with metal ions (e.g., heavy metals) through electrostatic interactions and complexation, affecting the bioavailability of these metals in the environment [22]. In this regard, HAs can be used in waste management or as a means of removing environmental pollution from such metals [23]. Motta et al. (2016) [24], used deprotonation followed by protonation as a method for the controlled production of HA nanoparticles that have a tendency to induce fungicidal effects, with the potential for developing new classes of cosmetics and pharmaceuticals [24].

The antiviral activity of HAs is also associated with a deprotonation process. As negatively charged polyanionic supramolecules, they could bind to positively charged viral glycoproteins, which would inhibit viral fusion with sensitive receptors on the cell membrane through a mechanism of competitive inhibition [25]. In vitro studies on various viruses are found in the literature: Herpes Simplex Virus (HSV) Type 1—HSV-1 [26,27], Herpes Simplex Virus Type 2—HSV-2 [27], Hepatitis B [28], Human Immunodeficiency Virus—HIV-1 [29,30,31], Coronavirus—(SARS-Cov) 2 [32,33] etc.

The process of OH/OOH deprotonation is also associated with the anti-inflammatory properties exhibited by HA [34]. The mechanism of action of HA as an anti-inflammatory agent can be explained by modulating cytokine production and inhibiting complement activation. HA can affect the production of inflammatory cytokines (interleukin (IL)-1β, IL-6 and tumor necrosis factor (TNF)-α), potentially reducing their levels and thus suppressing the inflammatory response [35]. HA can inhibit the activation of the complement system, the part of the immune system that is associated with inflammatory processes in the body [36]. Furthermore, HA can modulate the activity of immune cells such as neutrophils and lymphocytes and thus influence their inflammatory responses [37].

In addition to the presence of phenolic and carboxyl groups in the structure of HS, quinones have also been identified. Quinones are electron-accepting groups and are responsible for the production of reactive oxygen species (ROS). Nevertheless, the findings of Vašková et al. (2011) suggest no significant impact on antioxidant enzymes or on the reduction in redox potential [38]. This production of ROS is responsible for the bactericidal and fungicidal effects of HA, as well as its positive results in wound healing and in cancer therapy [34]. In addition to generating ROS, HA can also exert its bactericidal effect by disrupting the bacterial cell membrane. Disruption of the bacterial cell membrane results in damage to its integrity and function, leading to leakage of cellular contents and ultimately cell death. HS interact primarily with cell membranes, changing their permeability, while at the same time influencing the activity of enzymes that participate in biotransformation [39]. In vitro studies of humic preparations for efficacy against human pathogenic microorganisms have shown that they inhibit *Staphylococcus epidermidis*, *Staphylococcus aureus*, *Streptococcus pyogenes*, *Salmonella typhimurium*, *Proteus vulgaris*, *Enterobacter cloacae*, *Pseudomonas aeruginosa* and *Candida albicans*, but not *Enterococcus faecalis* and *Escherichia coli* with minimum inhibitory concentration (MIC) values generally in the range of 2.5–1.25 mg/mL, occasionally between 0.625 and 0.312between 0.625–0.312 mg/mL, and can be as low as 0.039 mg/mL when using synthetic HA. The origin and extraction method of natural HS determine their activity spectrum and intensity [40].

Nonetheless, HS’s involvement in ROS production is multifaceted, as it may also suppress ROS generation from alternative sources [41]. HS act as antioxidants due to the carboxyl, phenolic and polyphenolic hydroxyl functional groups they contain. These groups can release hydrogen radicals upon reaction with reactive species (e.g., ROS), resulting in more stable radicals. Furthermore, HS can inhibit ROS generation through metal chelation, modulate endogenous antioxidant enzyme activity and further support cellular redox balance [42,43,44]. HS can directly neutralize harmful ROS, such as superoxide radicals and hydroxyl radicals. For example, HAs obtained from various sources can scavenge O_2_^−^ up to 20% and −OH over 80% [38]. The antioxidant capacity (AOC) of HS, which differ significantly in structure, was determined using oxygen radical absorbance capacity (ORAC) assay. The determined AOC values are found to be close to the values for ascorbic acid and vitamin E. The results indicate that the AOC of HS is influenced by both phenolic and non-phenolic structural components, including carbohydrate-derived fragments. [45]. Volikov et al. (2021) [46] propose modification of the humic skeleton by incorporating redox groups with known redox properties to model the antioxidant properties of HA and FA. The results obtained show a significant difference in the redox and antioxidant properties of humic and FAs, with the parent HAs and their naphthoquinone derivatives showing high acceptor capacity, while FAs and their hydroquinone derivatives possess both high donor and high antioxidant capacity [46].

Overall, it can be inferred that HA is capable of generating ROS, particularly under UV irradiation, where it functions as both a photosensitizer and an electron carrier. This ROS generation represents a central mechanism underlying HA’s bioactivity and contributes significantly to its primary effects. Yet, HA’s role in ROS dynamics remains complex, as it may also act as an antioxidant, suppressing ROS formation depending on the sources from which it was obtained [47].

All of the aforementioned biomedical applications are discussed in more detail in the Section 5.

## 5. Biomedical Applications

**Figure 2:**
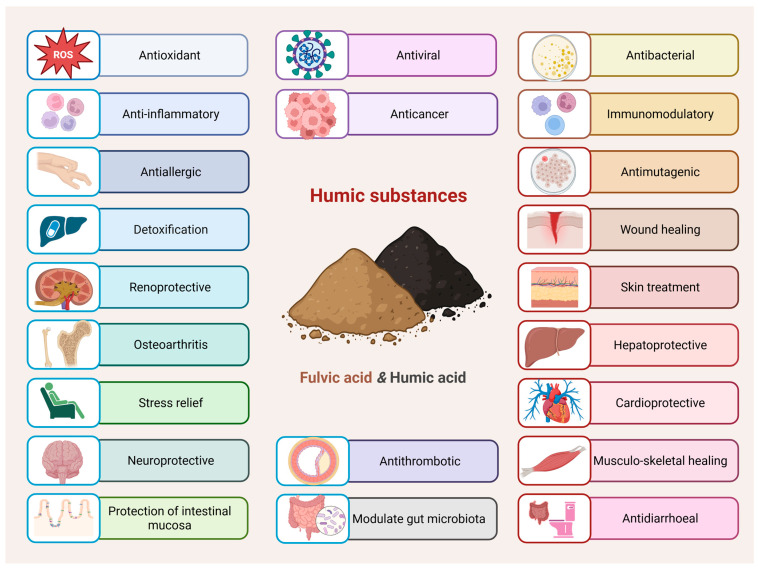
Biomedical applications of humic substances—while certain studies highlight specific effects for either HA or FA, both fractions have been reported to express most of the activities shown (Created in BioRender. https://BioRender.com/o8cgokg, accessed on 23 August 2025).

HS possess variable and undefined compositions that depend on their source, extraction method, and the types of functional groups they contain—such as carboxylic acids, quinones and phenols. Phenols and carboxylic acids, which deprotonate under neutral to alkaline conditions, are associated with additional functions, including the antioxidant and anti-inflammatory effects of HS. Notably, the phenolic groups are key contributors to the antioxidant activity of HS, owing to their ability to scavenge free radicals. Quinones contribute to the generation of ROS, which play a role in wound healing and exhibit antimicrobial (fungicidal and bactericidal) activity [34]. That is why over the years, HS have been extensively studied for their detoxifying properties, as well as their anti-inflammatory and immunomodulatory effects, anticancer, antimicrobial, and antiviral activities, as well as wound healing, antioxidant therapy and others (Figure 2) [48]. HS contain compounds with potentially significant pharmaceutical and medicinal properties, making the identification and investigation of these bioactive components highly valuable for pharmaceutical and biomedical applications [49].

### 5.1. Antioxidant Properties

**Figure 3:**
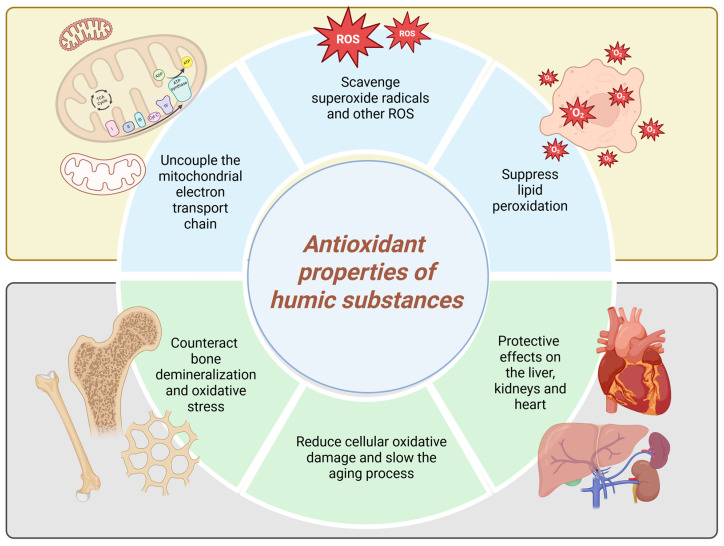
Antioxidant properties of humic substances (Created in BioRender. https://BioRender.com/84t0s0o, accessed on 23 August 2025).

Numerous studies have reported the antioxidant properties of HS [50], highlighting their protective roles, their ability to interrupt radical chain reactions, and their potential to prevent damage to cell membranes and biological macromolecules. Carbohydrates can make up as much as 10% of HS, potentially contributing to the overall antioxidant activity of these materials [45]. These exceptional properties of HS are presumed to contribute to their antioxidant activity in biological systems (Figure 3).

FA has been found to scavenge superoxide radicals and other ROS including superoxide, singlet oxygen, hydrogen peroxide, hydroxyl radicals, hypochlorous acid, and peroxynitrite extracellularly [51]. Intracellularly, however, FA can uncouple the mitochondrial electron transport chain in liver cells, a mechanism linked to reduced ROS generation [52]. An increase in antioxidant enzymes—such as glutathione, superoxide dismutase, and catalase—is commonly used as a biomarker to indicate the antioxidant activity of HS [51]. Klein et al., 2021 [45] showed that the ability of HS to scavenge peroxyl radicals is governed by the presence of both phenolic and non-phenolic structural components. They also highlighted the significant role of sugar moieties in contributing to the AOC of these materials [45].

Piotrowska and colleagues observed that peat-derived HS suppressed lipid peroxidation in human placental mitochondria, as evidenced by reduced levels of malondialdehyde [43]. The antioxidant properties of HS are believed to underlie their reported protective effects on the liver, nervous system, kidneys, and heart [45]. A study was conducted to investigate the efficacy of HA in terms of antioxidant and antiapoptotic effects in animal models compared with histopathological and neurological outcomes in hypoxic–ischemic brain injury. HA was found to reduce apoptosis and neuronal damage in rat brain tissue [53].

Antioxidants like HS have the potential to counteract bone demineralization and oxidative stress. In a 2025 study, Santos et al. investigated the impact of HA—sourced from vermicomposted agricultural biomass—on bone mineral content and oxidative stress markers in an experimental menopause model. Their findings showed that HA treatment enhanced bone elemental composition and regulated oxidative stress indicators in the gastrocnemius muscle, liver, and kidneys. These results could be a prerequisite for future clinical trials in humans [54]. Moreover, the antioxidant properties of HS also make them promising candidates for anti-aging therapies, as they help reduce cellular oxidative damage and thereby slow the aging process. This leads to improved vitality and overall enhancement of health in users [3].

### 5.2. Anti-Inflammatory and Immunomodulatory Effects

Synthetic low molecular weight HS (approximately 1500 Da) have been found to activate human neutrophils and contribute to pro-inflammatory responses [48]. However, HA-type compounds also demonstrate membrane-protective properties by inhibiting the lipoxygenase pathway within the arachidonic acid cascade—an essential component of cell membranes [32]. Additionally, another study reported that sodium humate significantly suppresses the formation of various types of edema [48].

Khuda et al. (2022) [55] reported that the anti-inflammatory effects of HS are enhanced in a dose-dependent manner through the inhibition of cyclooxygenase and lipoxygenase, as well as potentially through the suppression of other vasoactive compounds such as serotonin and histamine [55].

Multiple studies suggest that FA exhibits anti-inflammatory properties by inhibiting the release of proinflammatory mediators from immune cells [52,56,57,58]. FA extracts, derived from solubilized sludge, exhibit anti-inflammatory and anti-allergic effects by reducing β-hexosaminidase and histamine release in IgE-sensitized mast cells and basophils [52,58]. Yamada et al. 2007 [57] demonstrated that FA reduced the release of TNF-α, IL4, and IL13 from mast cells and proved its therapeutic potential in immune-related conditions such as eczema [57]. FA has been shown to suppress the expression of cyclooxygenase-2 and the secretion of prostaglandin E2 in primary human monocytes after stimulation with homocysteine [58]. Junek et al. (2009) [56] demonstrated that FA at a concentration of 200 μg/mL significantly reduced TNF-α expression in differentiated human monocytes following lipopolysaccharide exposure [56]. Furthermore, oral administration of carbohydrate-derived FA isolated from South Africa at a dose of 100 mg/kg has been shown to reduce paw edema in rats to a degree comparable to that of nonsteroidal anti-inflammatory drugs [59].

FA enemas have proven effective in treating chronic ulcerative colon infections. Additionally, the use of humic and fulvic substances in masks, poultices, and therapeutic baths has shown significant improvement in various skin conditions, including ulcers and other dermatological disorders [60]. Remarkably, fulvic/humic mineral baths have demonstrated up to a 90% success rate in treating ulcers, with benefits observed in both external and internal cases. Moreover, FA derived from Shilajit has exhibited anti-ulcerogenic potential in studies involving albino rats [61].

Along with everything listed so far, FA is classified as a natural health product and has been recognized for its therapeutic potential in managing diseases linked to chronic inflammation, including diabetes, cardiovascular conditions, and colitis. It also shows promise in alleviating inflammatory conditions of the cervix, such as cervical erosion, as well as reducing joint inflammation associated with rheumatoid arthritis. Additionally, HS have demonstrated the ability to bind to collagen fibers, supporting the repair and regeneration of damaged tendons and bone [51].

**Figure 4:**
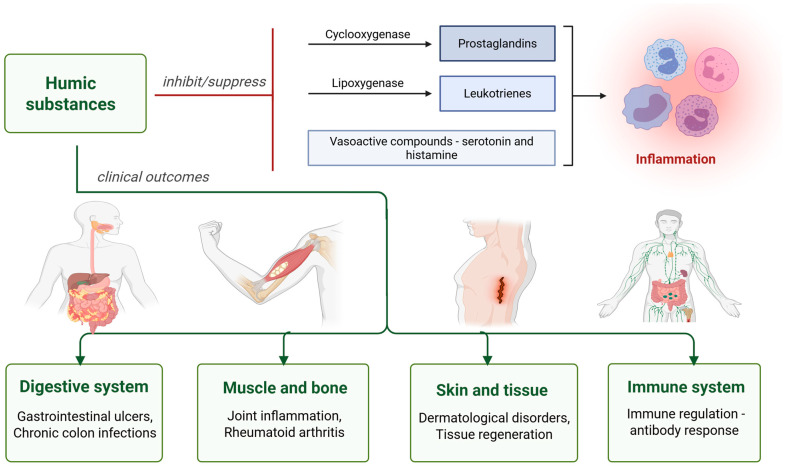
Anti-inflammatory and immunomodulatory effects of humic substances (Created in BioRender. https://BioRender.com/7gxpzy7, accessed on 23 August 2025).

HS have demonstrated positive effects on the immune system in both animals and humans. In the study by Trofimova et al. (2021) [36], HAs from oligotrophic Sphagnum magellanicum peat were found to suppress mitogen-induced production of the anti-inflammatory and to stimulate the production of proinflammatory cytokines. Moreover, repeated administration of humic acids (HAs) in mice was found to stimulate humoral immune responses, as indicated by a higher number of antibody-producing spleen cells and increased serum antibody titers after immunization with sheep red blood cells [36]. Humates can bind to sugars and facilitate the formation of complex saccharides, such as glycoproteins, which play a key role in modulating intercellular communication. These glycoproteins interact with T cells and natural killer cells, helping to maintain immune balance. Proper regulation is crucial, as an overabundance of T cells can contribute to autoimmune disorders, while excessive killer cell activity may lead to joint and bone damage, as seen in conditions like arthritis [51]. It could be concluded that HS exhibit notable immunomodulatory properties (Figure 4).

### 5.3. Anticancer Properties

HS are also known for their antimutagenic properties, acting as inhibitors of mutagenesis. They exert both antimutagenic activity—by blocking mutagenic processes within the cell—and desmutagenic activity—by preventing mutagenesis outside the cell. The extent of this biological activity depends on the concentration and chemical composition of the HAs, which vary based on their source, age, and environmental conditions during formation [34].

The biochemical and molecular effects of HS suggest that HS could serve as a strong candidate for cancer therapy. Despite considerable evidence supporting their beneficial impact on cancer treatment, these compounds have received relatively limited attention regarding their potential role in cancer etiology and other diseases [48]. A study examining the use of HS in traditional Persian, Arabic, Chinese, and Indian medicine [62] describes their anti-cancer properties, primarily attributed to their ability to protect cellular components—particularly DNA—from oxidative damage caused by superoxide anions and hydroxyl radicals. Furthermore, HAs have shown potential as supportive agents in cancer chemotherapy, where they help protect surrounding healthy tissue from oxidative stress induced by chemotherapeutic agents [3].

HS have demonstrated complete effectiveness in preventing esophageal tumors, achieving a 100% success rate. In the case of thyroid tumors, injections of HS have also shown high efficacy, with success rates reaching up to 90% [63]. These injections not only inhibited the growth of thyroid tumors but also significantly reduced their size. The anti-proliferative effects of HS human cervical cancer cells were confirmed in studies by Hseu et al. (2008) [64], while Aykac et al. (2018) [65] were the first to investigate the cytotoxic effects of HA on human breast adenocarcinoma cells.

Zolghadr et al. (2022) [66] reported that HA exhibited stronger anticancer and cytotoxic effects against breast cancer cell line MCF7 cells compared to FA. Their study demonstrated that both HA and FA could induce apoptosis and downregulate gene expression in treated MCF7 cells, with HA showing greater efficiency in triggering apoptosis. Furthermore, both HA and FA were found to enhance the elastic modulus and cell–cell adhesion forces in a dose-dependent manner. The model used in their analysis showed a high correlation between predicted and experimental % cell viability values, indicating its effectiveness in capturing the relationship between the treatment parameters and cellular response [66].

Yang et al. (2023) [67] investigated the anticancer effects of FA on pulmonary epithelial tumor cell lines (TC-1 cells). Their study confirmed that FA inhibited TC-1 cell proliferation in both a dose- and time-dependent manner. After 24 h of FA treatment, morphological changes such as reduced cell volume, loss of adherence, and an increased number of apoptotic cells were observed. FA also promoted DNA fragmentation, evidenced by the appearance of a DNA ladder pattern. Furthermore, FA treatment led to a decrease in the anti-apoptotic protein Bcl-2, suggesting activation of the intracellular apoptotic pathway. These findings support the conclusion that FA exerts anticancer effects through the induction of apoptosis in TC-1 cells [67].

### 5.4. Antibacterial, Antifungal and Antiviral Activities

FA has shown broad-spectrum antimicrobial properties against microorganisms typically present in the oral cavity. Studies suggest that FA can compromise microbial cell membranes, making it a potentially effective agent against a range of oral pathogens and biofilms [68]. Sherry et al. (2012) [69] demonstrated that FA exhibits antifungal activity against both planktonic and biofilm-associated *Candida albicans*, with effective concentrations of 0.125% and 0.25%, respectively. FA was found to be fungicidal, likely due to its ability to disrupt cell membrane function. Importantly, previous studies have reported no signs of toxicity in both rats and humans. In addition, FA has shown anti-inflammatory and wound healing properties, possibly through its free radical scavenging activity. While FA presents promising characteristics for use as an antiseptic, further research, including detailed cellular and in vivo studies, is needed to fully establish its therapeutic potential [69]. A study of revealed that HS isolated from Cretaceous shales in the Nowshera district of Pakistan exhibited strong antimicrobial properties. The extract showed significant antibacterial effects against *Salmonella typhi*, *Pseudomonas aeruginosa*, and *Escherichia coli*, with MICs of 0.82, 0.87, and 0.79 mg/mL, respectively. Marked inhibition was also recorded for *Bacillus subtilis* (MIC: 0.93 mg/mL) and *Staphylococcus aureus* (MIC: 1.12 mg/mL), when assessed in comparison with Imipenem as the reference drug. Furthermore, the HS extract demonstrated potent antifungal activity against *Alternaria alternata* and *Fusarium solani*, with MICs of 0.60 and 0.68 mg/mL, respectively. [55]. In the study by Yarkova et al. (2011) [70], the effects of sodium salts of indole-containing humic preparations on several bacterial species were investigated. Complete inhibition of *Staphylococcus aureus* and *Candida* colonies was observed, while the colony counts of *Escherichia coli* and *Salmonella enteritidis* were markedly reduced. Moreover, the colonies that did form were unusually small and dry, which is atypical for these microorganisms [70]. Verrillo et al. (2022) [71] reported that HS derived from composted artichoke and pepper residues showed strong antimicrobial activity against certain Gram-positive bacteria. Notably, *Staphylococcus aureus* exhibited inhibition zones of 10.1 mm and 9.4 mm, respectively, while *Enterococcus faecalis* demonstrated MIC values of 2.0 µg/mL and 14.0 µg/mL [71].

In the study by Vanimuthu et al. (2024) [72], three clinically relevant *Candida* species—*C. glabrata*, *C. tropicalis*, and *C. albicans*—were tested to evaluate the antifungal activity of HA. HA Extracts (100 µL) obtained from vermicompost, vermicast, and vermiwash demonstrated inhibition zones of 19 mm for *C. albicans*, 12 mm for *C. tropicalis*, and 15 mm for *C. glabrata*. These findings indicate that HA derived from vermiproducts may serve as promising antifungal therapeutic agents for the management of *Candida*-associated infections [72].

Dharejo et al. (2025) [73] investigated the antimicrobial potential of silver nanoparticles (AgNPs) conjugated with HA. Antibacterial activity and minimum bactericidal concentrations (MBC) were assessed using disk diffusion and broth dilution methods against *Staphylococcus aureus*, *Streptococcus pyogenes*, *Pseudomonas aeruginosa*, and *Escherichia coli* isolated from cow dung. The MBC values were 2.5 mg/mL for AgNPs alone and 5 mg/mL for AgNPs/HA. Notably, the AgNPs/HA conjugates exhibited enhanced inhibitory effects against all tested microorganisms [73].

Carboxylates and phenolates in the structure of HA facilitate electrostatic interactions with cationic domains of viral envelope glycoproteins. This interaction blocks the virus from fusing with host cell membrane receptors, primarily through a mechanism of competitive inhibition [25]. Notably, as early as 2001, Meerbach et al. [74] were among the first to associate the antiviral properties of HA according to its high content of carboxylic acid functional groups [74].

**Figure 5:**
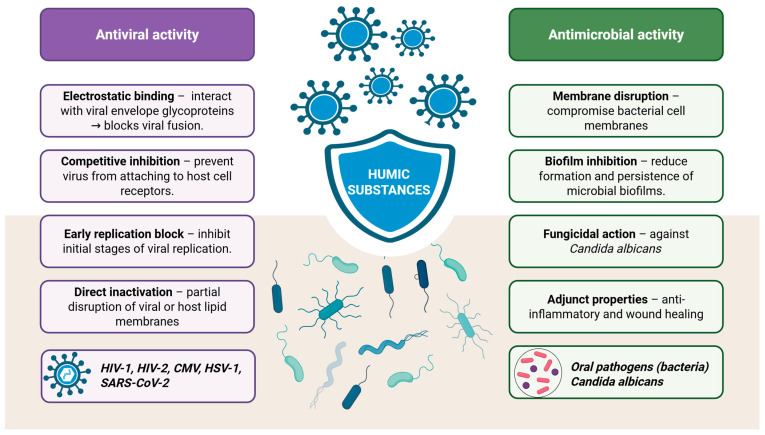
Antiviral and antimicrobial activity of humic substances (Created in BioRender. https://BioRender.com/qdesz4j, accessed on 23 August 2025).

As already said HA has demonstrated selective inhibitory effects against several human viruses, including human immunodeficiency virus types 1 and 2 (HIV-1 and HIV-2), cytomegalovirus (CMV), Coxsackie A9 virus, Herpes simplex virus type 1 (HSV-1) and the Vaccinia virus (Figure 5) [32,48]. HA molecules, which acquire a negative charge in neutral to basic environments, can suppress viral replication by binding to positively charged regions of the virus that are essential for its attachment to host cells [34]. Studies have shown that HS primarily inhibit the early stages of viral replication [32,48]. This antiviral activity has been particularly evident in the case of herpesviruses, where animal experiments confirmed the suppression of early replication by HA polymers [32].

In the study by Hajdrik et al. (2022) [33], the antiviral potential of a humic substance-based nutritional supplement—enriched with ascorbic acid, selenium, and zinc ions—was evaluated against the SARS-CoV-2 B.1.1.7 ‘Alpha Variant’ using a VeroE6 cell line. The results demonstrated a notable in vitro antiviral effect, with 50% inhibition of viral replication achieved at remarkably low concentrations of the active components [33].

Studies investigating the antiviral mechanism of HA have demonstrated that, for most viruses, their activity targets an early phase of viral replication. In research conducted by Wyde et al. [63] on SP-303—a HA-like polymer derived from a Euphorbiaceae shrub—it was found that SP-303 partially inactivates viruses through direct interaction with viral or host cell lipid membranes. Furthermore, clinical evidence suggests that dietary supplementation with FA has contributed to the resolution of severe viral respiratory infections commonly seen in children [75].

In a 2017 study, Zhernov et al. [6] explored the relationship between the structural characteristics of fractionated humic polyanions and their antiviral activity against laboratory strains of HIV-1. Utilizing a comprehensive HIV-1 replication model validated for antiviral screening, they confirmed the ability of all tested humic polyanions to inhibit HIV fusion. Notably, the most hydrophobic fractions also showed strong inhibition of HIV-1 reverse transcriptase. Based on their findings, the researchers concluded that these humic polyanions represent promising candidates for the development of affordable, multi-targeted microbicides with low toxicity, aimed at preventing HIV-1 transmission [6]. Additionally, HA synthesized from hydroquinone exhibited strong inhibitory effects against HIV-1 [32]. Among the various subclasses of HS, HA demonstrates superior antiviral efficacy compared to its lower-molecular-weight counterparts, such as FA [25].

The source of HA plays a significant role in determining its antiviral efficacy. According to Zhernov et al. (2021) [76], HA derived from coal demonstrates superior antiviral activity compared to that obtained from peat or synthetic analogs, such as caffeic acid and chlorogenic acid, when tested against specific viral targets [76]. Furthermore, earlier findings by Zhernov et al. (2018) [77] revealed a positive correlation between antiviral potency and both the carbohydrate content and lipophilicity of HA, suggesting that these molecular characteristics contribute critically to its bioactivity [77].

The observed antiviral activity of HS may result from the combined, synergistic effects of various components on the cell-virus interaction—such as antioxidant activity, inhibition of viral replication, strengthening of the cell membrane’s resistance, and suppression of pro-inflammatory cytokine release. To validate these findings, further in vivo challenge studies and well-designed clinical trials using candidate formulations containing HS are necessary [33].

### 5.5. Wound Healing Properties

The antioxidant properties of HS may support the wound healing process, which relies on oxygen and involves the generation of ROS during phagocytosis [3].

HA has also demonstrated anti-inflammatory effects [3,32,48] which may contribute to its wound healing capabilities across various tissue types as demonstrated in the study of Çalışır et al., (2018) [78]. This effect appears to be more pronounced during the later stages of healing, as HA-treated wounds exhibited significantly faster healing by the third week compared to those treated with chlorhexidine gluconate. Furthermore, histological analysis at the end of the three-week treatment period revealed that wounds treated with HA had smaller areas of inflammation than those in both the saline control and chlorhexidine-treated groups. HA treatment proved to be even more effective than chlorhexidine gluconate, a common agent used for managing oral wounds. Çalışır et al. demonstrated that HA promotes wound healing in the oral cavity, likely due to its combined antibacterial and anti-inflammatory properties [78].

In an animal study, Ji et al. (2016) [79] demonstrated that sodium humate enhances wound healing, likely through activation of the transforming growth factor beta (TGF-β) signaling pathway. Treated rat tissues also showed increased hydroxyproline levels and greater wound contraction during the healing phase. The authors conducted that Sodium humate could enhance wound healing in a rat model by accelerating wound contraction, elevating hydroxyproline levels, and promoting overall tissue repair [79]. In the presented study of Ji et al., [79] topical application of sodium humate significantly enhanced collagen production, as indicated by the markedly higher hydroxyproline content in the granulation tissue of the sodium humate-treated group compared to controls. Collagen, a major structural protein in connective tissue, is essential for wound healing [80]. The researchers concluded that the wound contraction and collagen synthesis observed with sodium humate treatment may be attributed to its ability to accelerate the overall wound healing process [79].

### 5.6. Detoxification (Adsorption Properties) and Heavy Metal Chelation

HS are known to possess a diverse array of functional groups, including carboxylic, phenolic, carbonyl, hydroxyl, amine, amide, and aliphatic moieties. This rich polyfunctionality makes HS some of the most effective natural chelating agents. When taken orally, HS act as natural chelating agents, offering an alternative approach to detoxification therapy. They bind to various harmful substances—such as heavy metals, pesticides, radioactive particles, and environmental carcinogens—thereby aiding in the detoxification of the body, liver, and digestive system [51]. Their zwitterionic nature enables interactions with both anions and cations—anions binding to positively charged groups and cations to negatively charged ones [81]. HS also possess the capacity to adsorb a wide range of organic compounds, including various toxins, pesticides, polycyclic aromatic hydrocarbons, and pharmaceuticals. Through this adsorption process, HA can partially neutralize these substances and support their elimination from the body [3]. The ability of HA to adsorb metals is significant not only from an environmental perspective but also in terms of their biological effects within the body. Their interaction with metal cations can influence various physiological processes, such as modulating metalloenzyme activity or aiding in the elimination of toxic metals during acute or chronic poisoning [82]. In cases of heavy metal intoxication, the capacity of HA to form complexes with these metals is particularly crucial, as it helps to neutralize their harmful effects. Two primary mechanisms of metal binding by HA have been identified: covalent bonding and coordination bonding, wherein the metal ion accepts an electron pair from the HA ligand [83].

Research has shown that HA is effective in removing botulinum neurotoxins in cases of sublethal chronic botulism in cattle [84], reducing lead accumulation and its toxic effects on the thyroid gland in hens [85], and decreasing levels of lead and cadmium in fish while simultaneously promoting improved growth rates [86].

### 5.7. Gut Microbiota Modulation

Swidsinski et al. (2017) [87] confirmed the traditional perspective that HAs act as broad-spectrum enhancers of microbial growth. Their study showed that HA supplementation led to a significant increase—over 30%—in the average concentration of the colonic microbiome (*p* < 0.001). Growth promotion was observed in 24 out of the 35 major bacterial groups analyzed. The only microbial groups negatively affected by HAs were Bacteroides (Bac303) and mycolic acid-containing Actinomycetes of the Mycobacterium subdivision (Myc657). All other groups either showed increased abundance or remained unaffected [87].

Given the evidence that HAs can enhance the concentration of colonic microbiota, a potential mechanism for their beneficial effects in alleviating irritable bowel syndrome (IBS) symptoms may lie in their prebiotic properties. Since dysbiosis is commonly observed in individuals with IBS, prebiotics are increasingly being explored as a promising avenue for future therapeutic development [88]. However, decreased diversity and reduced concentrations of colonic microbiota have been observed not only in inflammatory bowel disease (IBD) and IBS, but also in non-gastrointestinal conditions such as obesity, diabetes, rheumatism, and multiple sclerosis [89]. These alterations in the gut microbiome are increasingly believed to play a role in the pathogenesis of a wide range of diseases [87].

Another possible mode of action for HA involves their ability to protect the intestinal mucosa—potentially by forming a protective coating along the intestinal lining [78]. In their treatment of ulcerative colitis using FA, it is observed that FA formed a gelatinous protective layer over the ulcerated areas. This film appeared to reduce secretion and exudation from the wounds. The researchers attributed this effect to the colloidal nature and astringent properties of FA [90].

FA has been shown to possess significant antidiarrheal effects in both animals and humans and has been utilized in clinical practice for many years. However, due to its highly complex chemical composition and structure, the precise mechanisms underlying its antidiarrheal action remain incompletely understood. Research suggests that FA exhibits a dual mechanism of action on the intestinal mucosa: low molecular weight components (<5 kDa) primarily interact with the inner layers of the mucosa, while high-molecular-weight components (>5 kDa) act on the surface. Together, these components contribute synergistically to FA’s antidiarrheal efficacy [90]. One proposed antidiarrheal mechanism of FA involves its ability to protect the intestinal mucosa by preventing epithelial cell shedding and preserving the integrity of the intestinal lining. This effect is believed to result from its role in promoting epithelial cell maturation and enhancing collagen content within the intestinal tissue [91].

### 5.8. Antiallergic Properties

The antiallergic effects of HA are likely attributed to its ability to stabilize mast cell membranes, thereby preventing the pathological responses linked to the production and release of allergic mediators [75]. A clinical study of Snyman et al. (2002) [92] demonstrated that coal-derived FA at a <5% concentration significantly reduced wheal and flare size following allergen exposure in humans, with effects comparable to those of 1% hydrocortisone [92]. Additionally, FA derived from solubilized sludge and Canadian peat was found to suppress β-hexosaminidase and histamine release in IgE-sensitized mast cells and basophils, along with reductions in IL-4 and IL-13 levels [93]. These findings suggest that FA may possess notable anti-allergic properties [94].

### 5.9. Antihyperglycemia Activities by Type 2 Diabetes Mellitus

Type 2 Diabetes Mellitus is associated with chronic inflammation, oxidative stress, and alterations in the gut microbiome. It is characterized by impaired insulin signaling and reduced glucose uptake by cells, resulting in prolonged postprandial hyperglycemia. FA, particularly that extracted from Shilajit, has been shown to enhance superoxide dismutase activity in pancreatic beta cells. Studies conducted on diabetic rats have demonstrated its effectiveness in reducing hyperglycemia and alleviating related symptoms [48,52].

### 5.10. Neuroprotective Properties

The study of Özkan et al. (2015) [95] explored the neuroprotective potential of HA using a rat model of focal cerebral ischemia. The researchers employed a middle cerebral artery occlusion method and administered HA intraperitoneally after inducing ischemia. Biochemical analysis revealed a significant increase in superoxide dismutase and nuclear respiratory factor-1 levels in the HA-treated group compared to controls, while malondialdehyde levels—a marker of oxidative damage—were notably reduced. Histopathological evaluations showed reduced cerebral edema, vacuolization, neural degeneration, and tissue destruction in the HA group. These findings suggest that HA may mitigate cerebral ischemic damage by counteracting oxidative stress, indicating its potential as a therapeutic agent in ischemic brain injury [95].

### 5.11. Blood Properties

HA plays an active role in antithrombotic defense by stimulating the release of tissue-type plasminogen activator, which converts plasminogen into plasmin. This enzyme then breaks down insoluble fibrin into soluble fibrinogen degradation products. At the same time, HA inhibits thrombin—an essential coagulation enzyme—thereby suppressing the conversion of fibrinogen into fibrin monomers [48]. HA demonstrated a concentration-dependent ‘bimodal’ effect on blood coagulation, exerting opposing actions—either promoting or inhibiting clot formation—based on their dosage [96]. At low concentrations, it appears to promote clot formation by activating certain elements of the coagulation cascade. In contrast, at higher concentrations, HA has an inhibitory effect on coagulation, likely by disrupting or interfering with the same cascade processes [97].

FA, administered orally or via injection, has shown high efficacy in treating acute upper gastrointestinal bleeding, with a reported success rate of 95.6%. In China, specialized pharmaceutical formulations containing humic and FAs have been developed—backed by clinical studies—for the treatment of hemorrhoids [48].

## 6. Cytotoxicity of Humic Substances

The cytotoxic effects of HA, as part of the HS, are associated with the ability to induce apoptosis (programmed cell death) in certain cell lines, such as human vascular endothelial cells and breast cancer cells.

As already mentioned in a study by Zolghadr et al. (2022) [66], the effect of HA and FA on breast cancer cells (MCF7) was investigated. Cell viability was tested at different concentrations of HA and FA, including 10, 50, 100 and 200 μg/mL, respectively, for 14, 24 and 48 h. The results showed that HA and FA induced apoptosis, decreased cell viability and gene expression in solidified MCF7 cells, and a dose-dependent behavior of HA was observed in increasing the cell population in the Sub-G1 phase [66]. In another study, the cytotoxic effects of HA at concentrations of 5, 10, 20, 50, and 100 μg/mL in the human breast adenocarcinoma cell line MCF-7 for 24 and 48 h were investigated. Using the MTT method, the authors found that HA at a concentration of 100 μg/mL had a cytotoxic effect on the human breast adenocarcinoma cell line MCF-7 at both 24 and 48 h, with the effective dose of HA at the same time (24 and 48 h) being 50 μg/mL [65].

The cytotoxicity of HA obtained from peatlands in Central Kalimantan, Indonesia, on human vascular endothelial cells was investigated. The authors of the study found that 50 mg/L HA exhibited apoptosis-mediated cytotoxicity via the mitochondrial pathway, with this cytotoxicity being enhanced when endothelial cells were exposed to oxidative stress and reduced by the addition of vitamin C as an antioxidant. As a result of this study, long-term consumption of water obtained from the study area is not recommended, as it may cause endothelial cell damage and serious chronic health problems [98].

Another study demonstrated the synergistic effect of HA in enhancing Aβ-induced cytotoxicity in cultured human SK-N-MC neuronal cells. This enhancement of cytotoxicity was associated with activation of endoplasmic reticulum stress and mitochondrial dysfunction. These findings may provide new insights into the pathogenesis of Alzheimer’s disease, a neurodegenerative disorder associated with the accumulation of amyloid β peptide in the brain [99].

In a study by Yang et al. (2014) [100], prolonged (72 h) exposure to HA (25–200 µg/mL) induced cell cycle arrest and apoptosis in cultured RAW264.7 cells. Loss of cell viability, morphological changes, internucleosomal DNA fragmentation and cytochrome c release, activation of caspase-3 or caspase-9, and Bcl-2/Bax dysregulation were observed. HA also increased p53 expression, caused DNA damage, and may induce atherosclerosis through modulation of the macrophage-mediated immune system [100].

A study was also conducted to evaluate the genotoxic potential of HA using human peripheral blood lymphocytes. The results suggest that HA can induce genotoxicity in human lymphocytes due to DNA damage. HA-induced changes in Ca^2+^ homeostasis and ROS/RNS production are likely the main pathways for inducing genotoxicity, which could induce cancer by disrupting genetic integrity [64].

## 7. Drug Formulation and Drug Delivery

Research on HS, including HA and FA, continues to hold significant relevance in drug formulation and delivery systems. There are relatively few studies investigating HS as pharmaceutical excipients, primarily because HS are inherently complex materials. A typical HS sample contains a broad range of molecular sizes, and variations in their source lead to significant differences in chemical composition and structure. Moreover, HS can spontaneously alter their conformation and aggregation state in response to changes in solution conditions such as pH and ionic strength. In pharmaceutical science, even minor alterations in an excipient’s chemical composition are considered sufficient to classify it as a new excipient [101]. In drug delivery systems, HS serve as carriers that can enhance the bioactivity of therapeutic agents. They also contribute to improved solubility of various active compounds, such as andrographolide [48]. Additionally, complexation with HS has been shown to enhance the bioavailability and pharmacokinetic profile of drugs like carbamazepine, potentially facilitating more effective delivery to the brain [102].

Drug solubility is a critical factor in the development of effective drug delivery systems. Poor solubility often results in reduced bioavailability, ultimately compromising the therapeutic performance of the formulation. To compensate, higher doses of the active ingredient may be required, which can increase the risk of side effects and contribute to reduced patient compliance [103]. One key area of focus is their ability to form complexes with poorly soluble drugs, particularly those classified as BCS Class II and IV, thereby improving solubility and bioavailability. These macromolecules are believed to interact with such drugs primarily through complexation, offering a novel strategy to enhance drug performance [104].

The study by de Melo et al. (2016) [34] explored delivery systems derived from HS for incorporating poorly soluble active ingredients used in pharmaceutical, nutritional, and cosmetic applications. These systems involved the formation of complexes between HS and the active compounds through mechanisms such as hydrophobic interactions, covalent bonding, or chelation. The resulting complexes were shown to enhance solubility, permeability, and bioavailability, making them suitable for both topical and oral administration. Consequently, HA present a promising matrix for embedding bioactive ingredients within nano- or microstructured delivery systems [34].

Due to their macromolecular nature, negative charge, and the presence of multiple functional groups—such as carboxylic, amino, and phenolic moieties—FAs are capable of forming micelle-like structures, making them suitable for a range of applications beyond conventional use. Interestingly, formulations containing over 40% (*w*/*w*) FA have demonstrated significant mucoadhesivity, highlighting their potential in targeted drug delivery systems [105].

That is why FA has also been noted for its acid-buffering capacity and mucoadhesive properties. Given its favorable aqueous solubility profile, FA appears to have the greatest potential among HS for development as a pharmaceutical excipient. In contrast, HA, either alone or in combination with other pH-sensitive polymers, shows promise for use in enteric-coated drug delivery systems [101]. Several studies strongly support the use of FA as an effective water-soluble carrier. When formulated with aspirin, FA not only enhances the drug’s stability and bioavailability but also improves its anti-ulcerogenic effects compared to aspirin alone [106].

Khan et al. (2022) [107] developed a thymoquinone-loaded nanoemulgel using FA derived from peat to enhance solubility and absorption. The formulation demonstrated good pharmaco-technical properties and enhanced solubility, which significantly improved skin permeability in a mouse model. This led to increased localized bioavailability of thymoquinone, suggesting potential for clinical application in treating complex conditions like psoriasis. Enriched with the functional benefits of FA, the optimized formulation shows strong promise and merits further investigation in both preclinical and future clinical studies [107].

Konnova et al. (2023) [108] developed a transdermal patch formulation containing FA in an emulsion base, utilizing Pluronic Kolliphor^®^ P237 as a permeability enhancer. The patch was evaluated for its anti-inflammatory properties. Within seven days of treatment, FA administration led to normalization of leukocyte counts, C-reactive protein levels, and key biochemical markers of oxidative stress. Additionally, a significant reduction in edema was observed. The study demonstrated that FA influences the antioxidant defense enzyme system, helping to restore oxidative balance in a rat model of inflammation. These findings suggest that FA-based transdermal patches hold promise as a therapeutic option for managing inflammatory conditions associated with oxidative stress [108].

Savi et al. (2023) [109] synthesized novel humic–chitosan nanoconjugates (NCs) using HS extracted from three types of green compost: coffee husks, artichoke residues, and fennel residues. Solid-state NMR analysis revealed that HS from coffee husks and fennel residues were particularly rich in polysaccharidic and phenolic carbons, while HS from coffee husks showed a higher content of alkyl and carboxyl groups. The resulting NCs demonstrated antimicrobial activity against human pathogenic bacteria. This bioactivity was linked to their positive zeta potentials, which promoted adhesion to bacterial cell walls, along with their nanoscale size that facilitated cellular penetration and intracellular release of toxic components, thereby disrupting microbial biochemical processes. These findings highlight the potential of HS-based nanoconjugates as sustainable and effective antimicrobial agents [109].

Along with the ongoing research on HS for various biomedical applications, products such as dietary supplements and cosmetic products are also available and approved for use. A wide variety of products containing HS are available on the pharmaceutical and cosmetic market, some of which are summarized in Table 3.

## 8. Limitations in Using Humic Substances

Despite the many advantages and applications of HS, they also have some disadvantages. One of them is their significant compositional variability. This variation occurs not only between different categories—such as HAs, FAs, and HM—but also within the same type, influenced by their source and environmental conditions. Although some elemental ratios and functional groups remain relatively stable, the precise structure and concentration of these components can differ greatly, affecting their chemical behavior and biological activity [18]. The natural variability of HS makes standardization difficult, necessitating careful attention to their origin, the methods used for extraction, and the analytical techniques applied [125].

Toxicological assessments of FA suggest it has a generally favorable safety profile. Studies on acute, subchronic, and chronic toxicity—as well as evaluations of genotoxicity, developmental toxicity, and carcinogenicity—have consistently demonstrated good tolerance across a range of doses. Toxicological studies have shown that potassium humate is safe for human use at daily doses of up to 1 g per kilogram of body weight, while FA is considered safe at daily doses of up to 1.8 g per adult [126]. Although numerous in vitro studies have demonstrated that HS lack cytotoxic effects, some researchers emphasize the need for comprehensive preclinical evaluation before translating these findings into clinical application. This caution stems from concerns regarding potential limitations associated with HS, including limited bioavailability, the risk of heavy metal contamination, procoagulant effects, and the unintended chelation of essential trace elements [25].

## 9. Future Perspectives

The findings summarized in this review suggest that HS, including HA and FA, possess diverse biological activities. They may function as immune modulators, influence cellular redox balance, and contribute to gut health. HS have been shown to reduce proinflammatory markers, exhibit antimicrobial and antiviral effects, and mitigate oxidative stress. Additionally, they can induce apoptosis in various cancer cell models. HS also appear to influence the gut microbiome and may exert antithrombotic, antiallergic, antioxidant, cardioprotective, neuroprotective, hepatoprotective, and renoprotective effects. Collectively, these properties position HS as promising agents with therapeutic potential across a broad range of diseases and physiological conditions.

In the coming years, experimental studies on HS are expected to gain increasing importance and utility in the fields of drug formulation and delivery systems. Their diverse biochemical and molecular properties make them strong candidates for the treatment of various diseases, including cancer. While experimental studies remain crucial, molecular simulations of HS are expected to play an increasingly important role in the future. These simulations can enhance our understanding of experimental findings, aid in the design of targeted experiments, and help predict molecular-scale properties and interactions.

## 10. Conclusions

HS (HA and FA) are increasingly recognized as powerful, multifunctional soil biostimulants that enhance plant health, nutrient uptake, stress resilience, and carbon sequestration. Beyond agriculture, they display diverse biological activities, including antioxidant, anti-inflammatory, antimicrobial, antiviral, anticancer, and detoxifying effects. This broad spectrum of properties makes their future particularly promising, not only in traditional applications such as soil improvement and environmental remediation, but also in advanced fields such as drug formulation, targeted delivery systems, and therapeutic development. Ongoing research is focused on eco-friendly extraction technologies and the exploration of HS as pharmaceutical excipients or even active ingredients. Nevertheless, their complex and variable composition, dependence on source material, and potential safety risks emphasize the importance of standardization, along with comprehensive preclinical and clinical evaluation. In summary, HA and FA hold remarkable potential as natural therapeutics and biomedical excipients, but their safe and effective integration into medicine will depend on rigorous study and careful regulation.

## Figures and Tables

**Table 1:** Alkaline extraction process parameters.

Parameters	Alkaline Extraction of HS from Lignite Wastes	Alkaline Extraction of HS from Peat	Alkaline Extraction of HS from Corn Straw Compost
Alkaline solvent	NaOH	KOH	KOH
Temperature	85 °C	25 ± 2 °C	20 °C
Agitating speed	850 rpm	300 rpm	-
Time	4 h	4 h	24 h
Source	Sarlaki et al. (2019) [8]	Saito and Seckler (2014) [9]	Chi et al. (2023) [10]

**Table 2:** Physicochemical properties of HA and FA.

Physicochemical Characteristics	Humic Acid	Fulvic Acid
Colour	Brown-black	Yellow-brown
Molecular size Solubility Functional groups	10,000 Da–1,000,000 Da Insoluble < pH 2 Different proportion of carboxyl, phenolic hydroxyl, alcoholic hydroxyl, and carbonyl groups	1000 Da–10,000 Da Water soluble in all pH Different proportion of carboxyl, phenolic hydroxyl, alcoholic hydroxyl, and carbonyl groups

**Table 3:** Commercial dosage forms with HA and FA.

Name/ Active Ingredient	Dosage Form	Activities	Manufacturer
Acti Humic Acid/ Membrane active HA	Veggie capsules 350 mg	Functions as an immunomodulator; Helps eliminate harmful metals from the body; Regulates oxidative stress levels;	Herbamedica Bulgaria [110]
Dr. Mercola, Humic Fulvic Acid Complex/ HA and FA	Capsules 90 mg HA and 33 mg FA	Promotes gut health; Enhances energy levels; Neutralizes free radicals; Aids in detoxification	Dr. Mercola USA [111]
Humuszuur/ HA, FA and HM	Suspension content 5% HS	Provides antioxidant protection to guard cells from oxidative stress, damage, and premature aging; Strengthens the immune system, helping prevent colds and viral infections, while alleviating existing symptoms; Helps maintain the body’s acid–base balance; Supports brain function and enhances memory;	Mattisson Healthstyle Netherlands [112]
Humigold/ Humic and Fulvic minerals	Natural ionic plant-derived humic and fulvic powder	Can be applied as a paste when mixed with water; Aids in the natural healing of rashes; Helps draw out toxins from insect bites or poison ivy; Supports lymphatic detoxification when applied to underarms; Soothes and nourishes facial skin; Assists in healing acne;	ALIVE India [113]
HUMAC^®^ Nativ/ HA and HMs	Capsules 460 mg HA and 180 mg HM	Facilitates toxin absorption and removal; Exhibits anticarcinogenic properties; Demonstrates antioxidant activity;	HUMAC^®^ Slovakia [114]
HUMINIQUM/ HA and FA	Syrup 20 mg HA and 48 mg FA	Provides antioxidant effects; Exhibits antiviral properties; Supports hair health and helps reduce hair loss; Assists in managing obesity and fatigue; Enhances enzyme activity; Acts against anemia; Promotes recovery and regeneration after illnesses; Supports bone health and may help prevent osteoporosis;	HYMATO PRODUCTS Kft.—Hungary [115]
Fulvinezuur/ fermented FA	Suspension FA 6%	Supports normal liver function; Promotes healthy excretory system activity	Mattisson Healthstyle Netherlands [116]
Shilajit mumijo/ FA	Capsules 250 mg FA	Helps maintain normal blood sugar levels and lipid profile; Acts as a powerful adaptogen; Supports tissue regeneration; Exhibits anti-inflammatory and regenerative properties;	Vegavero International GmbH Germany [117]
Aviron rapid/ HA	Tablets 250 mg HA	Promotes respiratory system health and function; Supports the immune system; Maintains overall respiratory tract wellness;	Neopharm Bulgaria [118]
ACTIVOMIN/ HA	Capsules 400 mg natural HA	Helps relieve digestive issues, including gas and bloating; Supports regular bowel movements and alleviates constipation or diarrhea; Assists in recovery from food poisoning;	INSTITUT ALLERGOSAN Deutschland (privat) GmbH—Germany [119]
OMNi-LOGiC^®^HUMIN/ HA of type WH67^®^	Capsules 400 mg HA	Binds pollutants in the intestine and promotes their excretion through stool; Protects the intestinal mucosa; Soothes irritated nerve endings in the gut;	INSTITUT ALLERGOSAN Deutschland (privat) GmbH—Germany [120]
Humic Acid 50 mL Spray/HA	Spray 1 dose (6 injections, 1 mL): HA: 60 mg	Supports digestive health: reflux, gastritis, Helicobacter pylori, histamine and food intolerance; Supports immune and respiratory health: allergies, asthma; Supports skin health: shingles, psoriasis, eczema, atopic eczema, acne; Supports metabolic and systemic health: diabetes, arthritis, chronic fatigue; Helps reduce inflammation	ADVANCED HEALTH SK—Slovakia [121]
PURE&FULVIC Facial spray/FA	skin spray	Restores cellular balance; Provides a calming and soothing effect; Promotes purification and detoxification;	Humic Solution EU [122]
Fulvic Mist/ FA	Hair, skin and nail spray	Nourishes hair follicles; Reduces hair thinning and shedding; Alleviates scalp inflammation; Promotes hair growth; Removes harmful toxins to support keratin production and hair renewal;	Ful.Vic.Health UK [123]
Organic Fulvic Acid + 72 Trace Minerals/ FA and HA	Drops FA & HA 22 mg	Enhances mineral and nutrient transport, improving the bioavailability of vitamins and minerals; Supports cellular energy production, overall energy levels, and mental alertness; Acts as a natural free-radical scavenger and binds heavy metals and toxins, aiding detoxification; Promotes gut microbiome balance by supporting beneficial bacteria growth; Supports digestion, reduces bloating, and assists nutrient breakdown; Strengthens the body’s response to pathogens and exhibits antimicrobial properties	The Healthy Life 4 ME USA [124]

## Abbreviations

The following abbreviations are used in this manuscript:

HS	Humic substances
HA	Humic acid
FA	Fulvic acid
HM	Humin
HSV	Herpes Simplex Virus
HIV	Human Immunodeficiency Virus
SARS-Cov	Coronavirus 2019
TNF	Tumor necrosis factor
IL	Interleukin
ROS	Reactive oxygen species
AOK	Antioxidant capacity
ORAK	Oxygen radical absorbance capacity
CMV	Cytomegalovirus
TGF-β	Transforming growth factor beta
IBS	Irritable bowel syndrome
IBD	Irritable bowel disease
NCs	Nanoconjugates
MIC	Minimum inhibitory concentration
MBC	Minimum bactericidal concentrations
AgNPs	Silver nanoparticles
